# Altered Fecal Microbiota Correlated With Systemic Inflammation in Male Subjects With Methamphetamine Use Disorder

**DOI:** 10.3389/fcimb.2021.783917

**Published:** 2021-11-18

**Authors:** Di Deng, Hang Su, Yuehong Song, Tianzhen Chen, Qianqian Sun, Haifeng Jiang, Min Zhao

**Affiliations:** ^1^ Shanghai Mental Health Center, Shanghai Jiao Tong University School of Medicine, Shanghai, China; ^2^ Shanghai Key Laboratory of Psychotic Disorders, Shanghai, China; ^3^ CAS Center for Excellence in Brain Science and Intelligence Technology (CEBSIT), Chinese Academy of Sciences, Shanghai, China

**Keywords:** methamphetamine use disorder, gut microbiota, 16S rRNA sequence, systemic inflammation, gut-immune-brain

## Abstract

Methamphetamine use disorder (MUD) is a major public health problem worldwide with limited effective treatment options. Previous studies have reported methamphetamine-associated alterations in gut microbiota. A potential role of gut microbiota in regulating methamphetamine-induced brain dysfunction through interactions with the host immune system has been proposed, but evidence for this hypothesis is limited. The present study aimed to investigate the alterations in the fecal microbiota and explore its relationship with systemic inflammation in MUD. Fecal samples were obtained from 26 male subjects with MUD and 17 sex- and age- matched healthy controls. Fecal microbial profiles were analyzed by 16S rRNA sequencing. Plasma inflammatory markers were measured using enzyme-linked immunosorbent assay. Associations between fecal microbiota, systemic inflammatory markers and clinical characteristics were examined by Spearman partial correlation analysis while controlling for possible confounders. Compared with healthy controls, individuals with MUD showed no difference in fecal microbial diversity, but exhibited differences in the relative abundance of several microbial taxa. At the genus level, a higher abundance of *Collinsella*, *Odoribacter* and *Megasphaera* and lower levels of *Faecalibacterium*, *Blautia*, *Dorea* and *Streptococcus* were detected in subjects with MUD. More importantly, altered fecal microbiota was found to be correlated with plasma levels of CRP, IL-2, IL-6 and IL-10. The order *Lactobacillales*, exhibiting lower abundance in participants with MUD, was positively related to the duration of methamphetamine abstinence and the plasma level of anti-inflammatory cytokine IL-10. This study is the first to provide evidence for a link between altered fecal microbiota and systemic inflammation in MUD. Further elucidation of interactions between gut microbiota and the host immune system may be beneficial for the development of novel therapeutic approaches for MUD.

## Introduction

Methamphetamine (METH), an amphetamine-type stimulant, is the most commonly abused drug in China, with 1.18 million officially registered drug users reporting methamphetamine use, accounting for 55.5% of all drug users nationwide by the end of 2019 (Office of China National Narcotics Control Commission, 2020). Individuals with methamphetamine use disorder (MUD) often suffer from serious neuropsychological symptoms including psychosis, depression, anxiety and cognitive deficits, even after extended periods of abstinence (London et al., 2004; Paulus and Stewart, 2020). Despite of the adverse health consequences and economic burden ascribed to MUD (United Nations Office on Drugs and Crime, 2021), there are currently no FDA-approved pharmacological interventions available for treating MUD and limited evidence for the effectiveness of cognitive-behavioral therapy (Paulus and Stewart, 2020), highlighting the urgent need for more effective treatment options.

In recent years, there has been an increasing interest in the gut microbiota as a potential target for novel therapeutics, due to its tremendous impact on the brain, behavior, and health (Cryan et al., 2019). While the majority of work concerning the role of the gut microbiota in neuropsychiatric conditions has focused on autism spectrum disorder (Sharon et al., 2019; Wang et al., 2019), Alzheimer’s disease (Vogt et al., 2017; Bulgart et al., 2020), Parkinson’s disease (Petrov et al., 2017; Aho et al., 2019), depression (Bastiaanssen et al., 2020; Yang et al., 2020), anxiety (Jiang et al., 2018) and schizophrenia (Zhu et al., 2019; Golofast and Vales, 2020), the evidence of interactions between the gut microbiota and substance use has been relatively limited (Qin et al., 2021; Salavrakos et al., 2021), and even less is known in the context of METH use.

Evidence of METH-induced dysbiosis largely comes from observations using rodent models. Significant changes in the composition of gut microbiota have been found in rats and mice under different regimens of METH exposure (Ning et al., 2017; Angoa-Pérez et al., 2020; Chen et al., 2021; Forouzan et al., 2021; Wang et al., 2021). Further, the alteration of *Akkermansia* has been identified to be associated with the rat’s behavioral response to METH in a conditioned place preference (CPP) model (Yang C. et al., 2021), suggesting a possible role of gut microbiota in modulating METH-induced behavior and vulnerability to MUD. However, clinical evidence in relation to MUD remains scarce. So far, only two recently published clinical studies have addressed this issue (Cook et al., 2019; Yang Y. et al., 2021). A study among men who have sex with men (MSM) found that METH use in the past six months was associated with higher levels of pro-inflammatory bacteria and lower levels of butyrate-producing genera in rectal swab samples (Cook et al., 2019). While the study cohort was comprised entirely of MSM, all of whom were engaging in anal intercourse, findings from this study might not generalize to the heterosexual population since it has been suggested that sexual behavior might influence the composition of gut microbiota (Noguera-Julian et al., 2016). Another study involving 16 male methamphetamine abusers and 14 healthy subjects also reported alterations in gut microbiota diversity and composition associated with MUD (Yang Y. et al., 2021). However, this study was limited by a relatively small sample size and inablility to adjust for possible confounders, highlighting the need for additional investigation.

Multiple mechanisms, including immune, endocrine and neuronal pathways have been described to be involved in the microbiota-gut-brain signaling (Agirman and Hsiao, 2021). For MUD, the interaction between gut microbiota and the host immune system has been assumed to be one of the key mechanisms. It was hypothesized that METH-induced pro-inflammatory microbiota profile and gut barrier dysfunction might allow the translocation of bacteria and bacterial components into the circulation, thus exacerbate systemic inflammation, abnormal immune response and neuroinflammation, leading to aberrant brain function and behaviors (Prakash et al., 2017; Qin et al., 2021), but no definitive evidence currently exists to prove this hypothesis. Indeed, studies have shown that METH elicits dysregulation in the innate and adaptive immune response, causing changes to both pro- and anti- inflammatory cytokines (Papageorgiou et al., 2019). However, no previous study has investigated the possible connection between gut microbiota and systemic inflammation in MUD. Therefore, the present study is set out to characterize the gut microbiota in MUD and preliminary analyze the correlation between the altered gut microbiota, the markers of systemic inflammation and clinical features in MUD.

## Materials and Methods

### Study Subjects and Procedure

Individuals who met the Diagnostic and Statistical Manual of mental disorders, fifth edition (DSM-5) criteria for methamphetamine use disorder were recruited from Shanghai Compulsory Rehabilitation Center between November 2019 and October 2020. The diagnosis was made by two senior psychiatric doctors through face-to-face assessment. Subjects with other neurological or psychiatric disorders were ruled out. To limit confounding by the use of other illicit drugs such as heroin, cocaine and cannabis, individuals with other illicit substance use disorder within the past 5 years were also excluded. Healthy controls (HC) with no history of neuropsychiatric conditions or illicit substance use were recruited from the local community at the same time through advertisement and word-of-mouth. The inclusion criteria for all participants included age 18-65 years and body mass index (BMI) 18-35 kg/m^2^. For both groups, additional exclusion criteria were as follows: (1) chronic medical conditions such as autoimmune disease, diabetes, inflammatory bowel disease, liver cirrhosis and malignancy; (2) evidence of human immunodeficiency virus (HIV), syphilis, hepatitis B or C virus (HBV/HCV) infection; (3) use of antibiotics, probiotics, corticosteroids or any other immunomodulators within 3 months before sample collection; (4) history of gastrointestinal surgery within 5 years; (5) specific dietary habits such as high-fat diet preference or completely vegetable-based diet.

It is worth noting that individuals who drank alcohol or smoked cigarettes were not excluded from this study given the high prevalence of these conditions found in MUD (Rommel et al., 2015). Taking the effects of alcohol and nicotine on gut microbiota (Wannamethee et al., 2005; Leclercq et al., 2014; Ames et al., 2020) and systemic inflammation system (Wannamethee et al., 2005; Barr et al., 2016; Sureshchandra et al., 2019; Doggui et al., 2021) into consideration, these factors would be included as possible confounders in data analysis.

Of the 53 subjects initially screened, three participants did not meet the inclusion criteria, five individuals were excluded due to evidence of infection (one for HIV, one for HBV and three for HCV), and two people with diagnosis of diabetes were also excluded (**Supplementary Figure 1**). Ultimately, a total of 43 participants were included in this study. The study was approved by the Institutional Review Board and the Ethics Committee of Shanghai Mental Health Center and was conducted in accordance with the principles of the Declaration of Helsinki. Written informed consent was obtained from all participants, and all the private information of the subjects was kept confidential.

### Data Collection and Clinical Measurements

Demographic data and medical history were obtained from participants *via* a self-report questionnaire. Drug use history including age of first METH use, total years of METH use, abstinence time and frequency of METH use were also collected from subjects with MUD. METH craving was assessed by Visual Analog Scale (VAS), a 10-centimeter line ranging from 0 (no craving) to 10 (most intense craving). The subjects were requested to recall the last time they used METH when rating on VAS. For the measurement of alcohol and tobacco use, the Alcohol Use Disorders Identification Test (AUDIT; Saunders et al., 1993) and the Fagerstrom Test of Nicotine Dependence (FTND; Heatherton et al., 1991) were applied to all participants. Non-drinkers and non-smokers were rated as zero on AUDIT and FTND total score, respectively. Non-drinker was defined as those who never consumed alcohol or consumed less than one drink per month. Non-smoker was defined as having never smoked. To evaluate the severity of depression and anxiety in individuals with MUD, the Patient Health Questionnaire-9 (PHQ-9; Kroenke et al., 2001) and the General Anxiety Disorder Scale-7 (GAD-7; Spitzer et al., 2006) were used.

### Systemic Inflammatory Markers Analysis

Fasting venous blood was obtained from participants with MUD using heparinized disposable vacuum blood tubes and centrifuged within one hour of collection (4°C, 3000 rpm for 15 min) for plasma separation. Plasma was aliquoted and stored at -80°C until analysis. Five inflammatory markers in plasma were measured *via* sandwich enzyme-linked immunosorbent assay (ELISA) using commercially available kits (Beijing Rongxin Zhihe Biotechnology Co. Ltd., Beijing, China) according to the manufacturers’ protocols: C-reactive protein (CRP), tumor necrosis factor alpha (TNF-α), interleukin-2 (IL-2), interleukin-6 (IL-6) and interleukin-10 (IL-10). These markers of systemic inflammation were chosen base on published *in vivo* studies suggesting a possible biological association with METH use (Huckans et al., 2015; Papageorgiou et al., 2019). Laboratory personnel were blinded to the clinical data.

### Fecal Sample Collection, DNA Extraction, and 16S rRNA Sequencing

Fresh fecal samples were collected from all participants using sterile containers (SARSTEDT, Germany) preloaded with 5 mL fecal preservation solution (Realbio Genomics Institute, Shanghai, China) and stored at -80°C until processing. DNA extraction from fecal samples was performed following the manufacturer’s instruction of QIAamp Fast DNA Stool Mini Kit (Qiagen, Germany). The concentration of DNA was quantified with a NanoDrop 2000 spectrophotometer (Thermo Scientific, USA). DNA integrity was determined by 1% agarose gel electrophoresis. The V3-V4 region of the 16S rRNA gene was amplified with primers 341F (5’-CCTACGGGRSGCAGCAG-3’) and 806R (5’-GGACTACVVGGGTATCTAATC-3’) using KAPA HiFi Hotstart ReadyMix PCR Kit. Amplicons were gel purified, quantified and sequenced on the Illumina MiSeq PE250 sequencing platform (Realbio Genomics Institute, Shanghai, China).

### Sequence Data Processing and Bioinformatic Analysis

The paired-end sequencing reads were assembled by PandaSeq v2.9 (Masella et al., 2012). Reads containing poly-N and low-quality reads were removed from the raw data, and only the reads of length between 250-500 nt were obtained as clean reads. Clean reads were clustered into operational taxonomic units (OTUs) based on 97% similarities using Usearch v7.0.1090 (Edgar, 2013). Taxonomy was assigned to representative OTU sequences by the RDP classifier based on the RDP database of release 11 (http://rdp.cme.msu.edu; (Wang et al., 2007; Cole et al., 2014). The resulting OTU table was normalized by converting read counts into relative abundances. Unclassified taxa at a given taxonomic level were excluded from analysis.

Alpha diversity and beta diversity were calculated based on rarefied OTU counts in QIIME v1.9.1 (Caporaso et al., 2010). Alpha-diversity indices used included Chao1, observed species, Shannon index and Simpson index. The two-sided wilcox.test function in R Statistical Software v4.1.0 (R Core Team, 2021) was used to compare each α-diversity index between the MUD and HC groups. Beta-diversity was estimated by computing weighted UniFrac (Lozupone et al., 2007), unweighed UniFrac (Lozupone and Knight, 2005), Bray-Curtis and Jaccard distance matrices. Differences in beta diversity metrics were tested by permutational multivariate analysis of variance (PERMANOVA) using the adonis function with 999 permutations and visualized with Principal Coordinate Analysis (PCoA) using Vegan R package (v2.5-7; https://CRAN.R-project.org/package=vegan).

The linear discriminant analysis (LDA) effect size (LEfSe), an algorithm that emphasizes both statistical significance and biological relevance (Segata et al., 2011), was applied on the online interface Galaxy (https://huttenhower.sph.harvard.edu/galaxy/) to identify differentially abundant taxa (LDA score > 2.0, p < 0.05) between the MUD and HC groups. Similarly, MaAsLin2 (multivariate analysis by linear models) package in R was used to detect specific associations between microbial community abundance and clinical phenotype while de-confounding the effects of any other confounders (Mallick et al., 2021). MaAsLin2 was performed while adjusting for covariates of age, BMI, AUDIT total score, and FTND total score. The relative abundance of taxa was arc sine square root transformed (transform = AST). Any taxon-level association with a Benjamini-Hochberg false detection rate (FDR)-corrected q-value < 0.25 was considered statistically significant, which is the default threshold for significance in MaAsLin2 and is commonly used in microbiome studies (Lim et al., 2016; Leung et al., 2020). To decrease the noise, low-abundance taxa (average relative abundance < 0.01%) and taxa detected in < 10% of all samples were eliminated before the LEfSe and MaAsLin2 analyses.

For functional inferences of the microbial communities, the Kyoto Encyclopedia of Genes and Genomes (KEGG; Kanehisa et al., 2016) was used to define orthologous gene functions in terms of KEGG Orthology (KO) groups. KO abundances were predicted using PICRUSt v1.0.0 (Phylogenetic Investigation of Communities by Reconstruction of Unobserved States; Langille et al., 2013) tables to first normalize each OTU’s relative abundance by its 16S rRNA copy number and then infer KO abundance from the genomic content of each OTU. Differentially abundant KOs between the MUD and HC groups were identified by Wilcoxon rank sum test. All comparisons were corrected for multiple testing (Benjamini–Hochberg method, FDR q < 0.05).

### Statistical Analysis

Comparisons of demographic and clinical information between the MUD and HC groups were analyzed using SPSS software (v26.0, IBM Statistics, Chicago, IL, USA). Continuous variables were tested using Student’s t test or Mann-Whitney U test depending on the distribution normality evaluated by Shapiro-Wilk’s test. Categorical variables were examined by Pearson Chi-square test or Fisher’s exact test. P value (two-sided) < 0.05 was considered significant.

Spearman partial correlation analyses were run to investigate the associations between the fecal microbiota, clinical variables, and systemic inflammation in the MUD group while controlling for age, BMI, AUDIT score, and FTND score. Correlation coefficients (r) and corresponding two-sided p values were computed using the ppcor R package (v1.1; https://CRAN.R-project.org/package=ppcor). P values were adjusted for FDR by the Benjamini-Hochberg method using the R function p.adjust. Statistically significant correlations (FDR q value < 0.05) were used to construct the correlation networks, which were visualized *via* a Circos image utilizing Circlize R package (Gu et al., 2014).

Other figures were produced using VennDiagram R package (v1.6.20; https://CRAN.Rproject.org/package=VennDiagram), ggplot2 R package (Wickham, 2016), and also Graph Pad Prism9 (Graph Pad Software, http://www.graphpad.com).

## Results

### Demographic and Clinical Characteristics of Participants

Twenty-six subjects with MUD and 17 healthy individuals were enrolled in this study. All participants were male. All of the subjects belonged to the same nationality (Han Chinese) and lived in Shanghai city. Demographic and clinical characteristics were listed in **Table 1**. There were no significant differences between the MUD and HC groups in terms of age, BMI and years of education (all p > 0.05). However, a greater proportion of individuals with MUD were not married (p = 0.036). As expected, subjects with MUD had significantly higher AUDIT and FTND total score than the healthy control individuals (both p < 0.001). In the MUD group, the average age of first METH use was 27.47 years old, the median duration of METH use was 4 years, and the median duration of abstinence was 8.22 months.

**Table 1:** Demographic and clinical characteristics of study participants.

Characteristics	MUD group (N = 26)	HC group (N = 17)	P-value
Age, years	33.34 [31.80-38.17]	34.00 [26.5-55.5]	0.619^a^
BMI, kg/m^2^	24.92 ± 3.06	23.63 ± 3.73	0.222^b^
Education, years	12 [9-14.25]	16 [9-17]	0.188^a^
Married, N (%)	7 (26.9%)	10 (58.8%)	0.036^c^
Employed, N (%)	21 (80.8%)	17 (100%)	0.139^d^
AUDIT	9 [0-13.25]	0 [0-0]	<0.001^a^
FTND	5 [2.75-6]	0 [0-1]	<0.001^a^
**METH use characteristics**			
Age of first use	27.47 ± 5.33	NA	NA
Total years of use	4 [3-7]	NA	NA
Months of abstinence	8.22 [3.38-16.04]	NA	NA
Frequency of use (in last month before rehabilitation)			NA
Almost everyday	0 (0%)	–	
3-5 times a week	4 (15.4%)	–	
1-2 times a week	8 (30.8%)	–	
Less than once a week	14 (53.8%)	–	
Craving (VAS)	2 [0.5-4.35]	–	
**Emotion**			
PHQ-9	3 [0.75-5]	–	NA
GAD-7	1 [0-4.25]	–	NA
**Inflammatory markers**			
CRP, mg/L	7.72 ± 1.66	–	NA
TNF-α, pg/mL	69.88 ± 12.11	–	NA
IL-2, ng/mL	6.84 ± 1.50	–	NA
IL-6, pg/mL	27.01 ± 7.41	–	NA
IL-10, pg/mL	937.21 ± 169.26	–	NA

Data are presented as mean ± standard deviation (SD), Median [IQR, inter-quartile range], or N (%). ^a^p values based on Mann-Whitney U test; ^b^p values based on Student’s t test; ^c^p values based on Pearson chi-square test; ^d^p values based on Fisher exact test. MUD, methamphetamine use disorder; HC, healthy control; BMI, body mass index, calculated as weight in kilograms divided by height in meters squared; AUDIT= Alcohol Use Disorders Identification Test; FTND, Fagerstrom Test for Nicotine Dependence; NA, not applicable; VAS, Visual Analogue Scale; PHQ-9, Patient Health Questionnaire-9; GAD-7, General Anxiety Disorder Scale-7; CRP, C-reactive protein; TNF-α, Tumor necrosis factor-alpha; IL, Interleukin.

### Unaltered Overall Structure of the Fecal Microbial Communities in MUD

In the current study, 16S rRNA gene amplicon sequencing of the fecal samples obtained from 43 participants generated 1,564,800 clean reads, ranging from 28,213 to 38,982 and with a mean of 36,391 reads. A Venn diagram was drawn to display the shared and distinct OTUs between the MUD and HC groups. In total, 418 OTUs were shared in both groups, while 236 were unique for the MUD group, and 78 were specific for the HC group (**Supplementary Figure 2A**). No significant differences in alpha diversity were identified between the two groups when measured by four different indices (Chao1, observed species, Shannon or Simpson index; all p > 0.05; **Supplementary Figure 2B**). Beta diversity according to the Bray-Curtis, Jaccard, unweighted UniFrac and weighted UniFrac distances showed no differences in the microbial composition between the groups (PERMANOVA, all p > 0.05), with no evidence of a clear separation in PCoA representations (**Supplementary Figure 3**). Compositional analysis revealed that *Bacteroidetes*, *Firmicutes*, *Proteobacteria* and *Fusobacteria* were the most abundant phyla in our sample of 43 subjects (**Supplementary Figure 4A**). At the genus level, *Bacteroides*, *Prevotella*, *Faecalibacterium*, *Megamonas* and *Roseburia* were among the major phylotypes in both groups (**Supplementary Figure 4E**).

### Identification of Differentially Abundant Taxa Between MUD and HC

**Figure 1:**
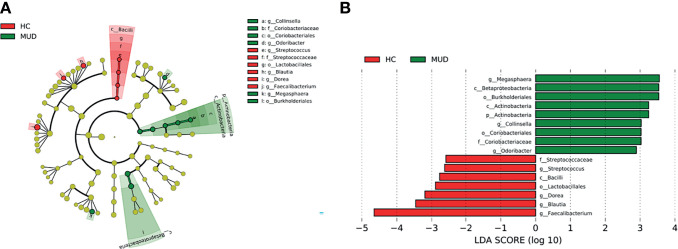
Taxa with significant different abundance between the MUD and HC groups. **(A)** Taxonomic cladogram obtained from LEfSe analysis displaying differentially enriched bacterial taxa in HC (red) and MUD (green). **(B)** Linear discriminant analysis (LDA) revealed the effect size of each differentially abundant taxa (LDA score > 2, p < 0.05).

**Figure 2:**
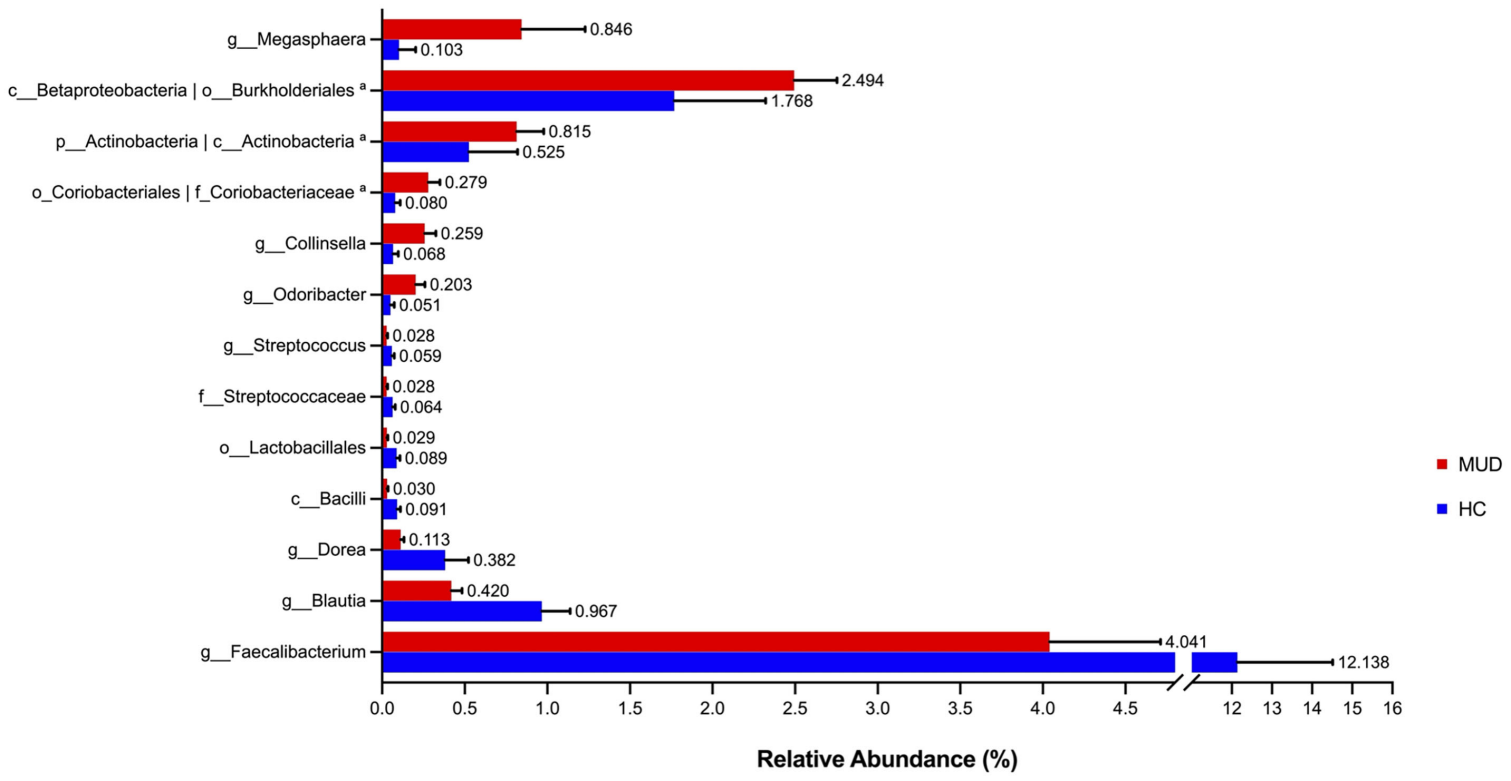
Barplots for relative abundance of significantly different taxa identified by LEfSe in the two groups. The x-axis represents the means proportion of the taxa. Each bar shows mean ± SEM. ^a^The taxa showed the same relative abundance at both taxa levels.

Despite the overall similarity of microbial structure between the MUD and HC groups, application of the LEfSe method identified several taxa with significant different abundance between the two groups (LDA score >2, p <0.05; **Figures 1A, B**). Compared to the HC group, the relative abundance of *Actinobacteria* at the phylum level; *Betaproteobacteria* and *Actinobacteria* at the class level; *Burkholderiales* and *Coriobacteriales* at the order level; *Coriobacteriaceae* at the family level; *Megasphaera*, *Conllinsella* and *Odoribacter* at the genus level were significantly higher in the MUD group. Conversely, the HC group was significantly enriched with the class *Bacilli*, the order *Lactobacillales*, the family *Streptococcaceae*, and the genera *Faecalibacterium*, *Blautia*, *Dorea* and *Streptococcus*. The relative abundance of taxa with significantly different abundance in the two groups were shown (**Figure 2**).

To further verify the changes in the microbial components, a multivariate regression model, MaAsLin2 was performed. The enrichment of the genus *Conllinsella* (coefficient = 0.0664, FDR q = 0.0002) together with upper-level taxa such as the family *Coriobacterianceae* (coefficient = 0.0575, FDR q < 0.0001), and the genus *Odoribacter* (coefficient = 0.0474, FDR q = 0.1328) in the MUD group was confirmed by MaAsLin2 even after adjusting for the covariates of age, BMI, AUDIT score, and FTND score, showing strongly positive associations with MUD (Model 2, **Table 2**). Consistent with the LEfSe results, the genera *Faecalibacterium*, *Blautia*, *Dorea*, and *Streptococcus* were found to be negatively associated with MUD, whereas the order *Burkholderiales* and upper-level taxon *Betaproteobacteria* had positive associations with MUD in the crude model (all FDR q < 0.25; Model 1, **Table 2**). However, these associations were no longer significant after adjustment for confounders (all FDR q > 0.25; Model 2, **Table 2**). In addition, MaAsLin2 detected the genus *Clostridium sensu stricto*, which was not identified in the LEfSe analysis, being negatively associated with MUD although the association in the adjusted model did not remain statistically significant (FDR q > 0.25; Model 2, **Table 2**). Analysis using MaAsLin2 showed that most significant features identified by LEfSe remained significant after adjustment for age, BMI, AUDIT score, and FTND score. The concordance of results from two different statistical methods provided confidence that the differences observed are true associations rather than false positives.

**Table 2:** Detection of taxa significantly associated with MUD by MaAsLin2.

Taxa	Model 1 (unadjusted)			Model 2 (adjusted for age, BMI, AUDIT score, and FTND score)		
	Coef.^#^	p value	q value	Coef.^#^	p value	q value
**p:Actinobacteria**	0.0254	0.1014	0.6085	0.0626	0.0060	0.1096*
**p:Firmicutes; c:Bacilli**	-0.0135	0.0002	0.0018**	-0.0134	0.0128	0.2345*
p:Proteobacteria;c:Betaproteobacteria	0.0386	0.0346	0.1903*	0.0207	0.4583	0.7180
**p:Actinobacteria; c:Actinobacteria**	0.0255	0.1005	0.2762	0.0629	0.0058	0.1592*
**p:Firmicutes; c:Bacilli; o:Lactobacillales**	-0.0138	0.0002	0.0021**	-0.0139	0.0107	0.1387*
**p:Actinobacteria; c:Actinobacteria; o:Coriobacteriales**	0.0215	0.0123	0.0796*	0.0557	5.94E-07	3.86E-05**
p:Proteobacteria; c:Betaproteobacteria; o:Burkholderiales	0.0386	0.0345	0.1496*	0.0208	0.4569	0.6906
p:Firmicutes; c:Bacilli; o:Lactobacillales; f:Streptococcaceae	-0.0098	0.0047	0.0858*	-0.0110	0.0312	0.4957
**p:Actinobacteria; c:Actinobacteria; o:Coriobacteriales; f:Coriobacteriaceae**	0.0220	0.0127	0.0858*	0.0575	5.52E-07	5.52E-05**
p:Firmicutes; c:Clostridia; o:Clostridiales; f:Clostridiaceae1	-0.0290	0.0129	0.0858*	-0.0322	0.0651	0.5099
p:Firmicutes; c:Clostridia; o:Clostridiales; f:Lachnospiraceae; g:Blautia	-0.0338	0.0011	0.0539*	-0.0237	0.1234	0.6760
p:Firmicutes; c:Clostridia; o:Clostridiales; f:Ruminococcaceae; g:Faecalibacterium	-0.1369	0.0049	0.1045*	-0.0874	0.2228	0.6853
**p:Actinobacteria; c:Actinobacteria; o:Coriobacteriales; f:Coriobacteriaceae; g:Collinsella**	0.0275	0.0061	0.1045*	0.0664	6.11E-07	0.0002**
p:Firmicutes; c:Clostridia; o:Clostridiales; f:Clostridiaceae1; g:Clostridium sensu stricto	-0.0317	0.0127	0.1625*	-0.0365	0.0579	0.5473
**p:Bacteroidetes; c:Bacteroidia; o:Bacteroidales; f:Porphyromonadaceae; g:Odoribacter**	0.0223	0.0232	0.2202*	0.0474	0.0016	0.1328*
p:Firmicutes; c:Clostridia; o:Clostridiales; f:Lachnospiraceae; g:Dorea	-0.0207	0.0295	0.2202*	-0.0147	0.3240	0.7311
p:Firmicutes; c:Bacilli; o:Lactobacillales; f:Streptococcaceae; g:Streptococcus	-0.0084	0.0302	0.2202*	-0.0083	0.1406	0.6766

^#^Coefficients from the generalized linear model using MaAsLin2 on pairwise testing between the two groups. *q < 0.25; **q < 0.05; q values were calculated using Benjamin-Hochberg correction (FDR). In Model 2, taxa with significant difference and their q values were bolded. p, phylum; c, class; o, order; f, family; g, genus.

### Functional Properties of Fecal Microbiota Predicted by PICRUSt

PICRUSt based on closed-reference OTU was used to predict the abundance of functional categories. A total of 14 KOs were identified with significantly different abundance in the fecal microbiota between the MUD and HC groups (all FDR q < 0.05; **Supplementary Figure 5**). Interestingly, all of the identified KOs were enriched in the HC group, including purine metabolism related genes such as K16839, K16840 and K16842, fatty acid metabolism and biosynthesis related gene KO18743, and quorum sensing related gene KO13816.

### Relationships Between Altered Fecal Microbiota, Clinical Characteristics and Systemic Inflammation in MUD

**Figure 3:**
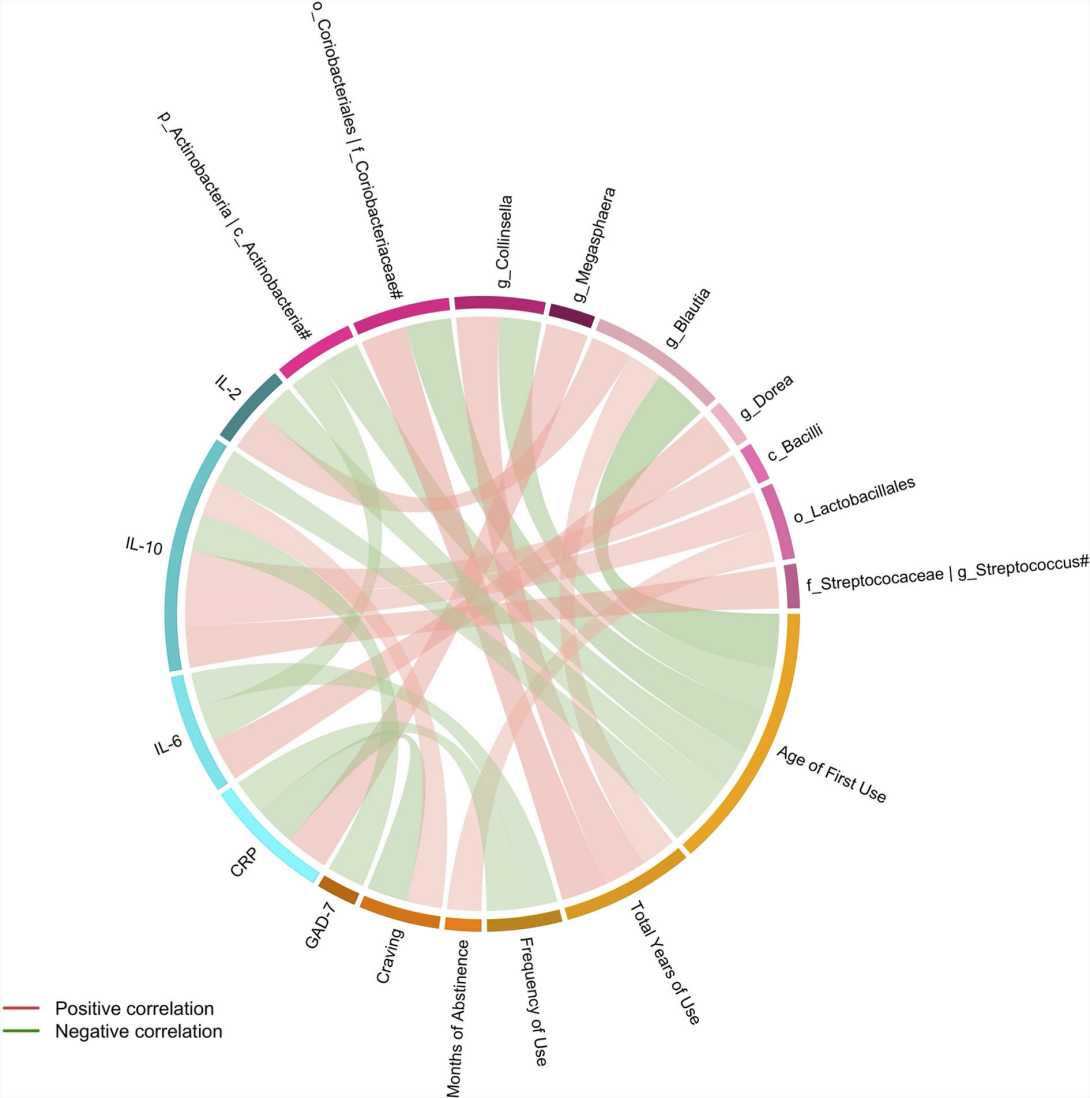
Connections between altered fecal microbiota, clinical variables and systemic inflammation identified by Spearman partial correlation. Adjusted for age, BMI, AUDIT score, and FTND score. Only significant correlations with an FDR q value < 0.05 were shown. ^#^The taxa showed the same relative abundance at both taxa levels. c, class; o, order; f, family; g, genus; CRP, C-reactive protein; IL, Interleukin; GAD-7, General Anxiety Disorder Scale-7.

Spearman partial correlation analysis was conducted to explore the relationships between the altered fecal microbial taxa obtained by LEfSe, clinical characteristics, and markers of systemic inflammation in MUD (N = 26), controlling for age, BMI, AUDIT score, and FTND score. Significant correlations were identified between twelve microbial taxa, six clinical variables, and four systemic inflammatory factors (**Table 3** and **Figure 3**). The relative abundance of the genus *Collinsella* and upper-level taxon *Coriobacteriaceae*, was positively related to the total years of METH use (r = 0.5312, FDR q = 0.0390; r =0.5666, FDR q = 0.0390 respectively). Besides, the order *Lactobacillales* showed significantly positive correlations with the months of METH abstinence (r = 0.4233, FDR q = 0.0496). The class *Bacilli* and sub-taxa were found to be positively correlated with the level of anti-inflammatory cytokine IL-10. Moderate correlations (|r| = 0.4-0.6, all FDR q < 0.05) were also observed for the class *Actinobacteria* and the genus *Dorea* with IL-6, the genus *Blautia* with IL-2, and the genus *Megasphaera* with CRP. Regarding the relationships between clinical measurements and systemic inflammation, we found the severity of anxiety evaluated by GAD-7 showing a negative correlation with IL-10 (r = -0.4667, FDR q = 0.0472). Surprisingly, craving was negatively associated with CRP (r = -0.5003, FDR q = 0.0390) while positively associated with IL-10 (r = 0.4408, FDR q = 0.0472). Moreover, frequency of METH use had moderately negative correlations with CRP (r = -0.4438, FDR q = 0.0472) and IL-6 (r = -0.4270, FDR q = 0.0496).

**Table 3:** Correlations between altered fecal microbiota, clinical variables and markers of systemic inflammation. .

Interactions between variables		Correlation coefficient (r)	p value	q value
**Fecal Microbiota × Clinical Variables**				
Age of First Use	p:Actinobacteria; c:Actinobacteria^#^	-0.4853	0.0221	**0.0441**
	o:Coriobacteriales; f:Coriobacteriaceae^#^	-0.5638	0.0063	**0.0390**
	g:Collinsella	-0.5181	0.0135	**0.0390**
	g:Blautia	-0.6656	0.0007	**0.0159**
Total Years of Use	o:Coriobacteriales; f:Coriobacteriaceae^#^	0.5666	0.0060	**0.0390**
	g:Collinsella	0.5312	0.0110	**0.0390**
	g:Blautia	0.4395	0.0407	**0.0472**
Months of Abstinence	o:Lactobacillales	0.4233	0.0496	**0.0496**
**Fecal Microbiota × Systemic Inflammation**				
CRP	g:Megasphaera	0.5221	0.0127	**0.0390**
IL-6	p:Actinobacteria; c:Actinobacteria^#^	-0.4501	0.0356	**0.0472**
	g:Dorea	0.5244	0.0122	**0.0390**
IL-10	c:Bacilli	0.4529	0.0343	**0.0472**
	o:Lactobacillales	0.4660	0.0288	**0.0472**
	f:Streptococaceae; g:Streptococcus^#^	0.5137	0.0145	**0.0390**
IL-2	g:Blautia	0.5069	0.0161	**0.0390**
**Systemic Inflammation × Clinical Variables**				
Age of First Use	IL-2	-0.4394	0.0408	**0.0472**
	IL-10	-0.4252	0.0485	**0.0496**
Frequency of Use	CRP	-0.4438	0.0385	**0.0472**
	IL-6	-0.4270	0.0475	**0.0496**
Craving	CRP	-0.5003	0.0177	**0.0390**
	IL-10	0.4408	0.0401	**0.0472**
GAD-7	IL-10	-0.4667	0.0285	**0.0472**

Spearman partial correlation adjusting for age, BMI, AUDIT score, and FTND score (N =26). Q values were calculated using Benjamin-Hochberg correction (FDR); q values < 0.05 were bolded. ^#^The taxa showed the same relative abundance at both taxa levels. c, class; o, order; f, family; g, genus; CRP, C-reactive protein; IL, Interleukin; GAD-7, General Anxiety Disorder Scale-7.

## Discussion

To the best of our knowledge, the current study is the first to provide evidence for a link between altered fecal microbiota and systemic inflammation in MUD. Consistent with previous studies (Cook et al., 2019; Yang Y. et al., 2021), our results demonstrated that subjects with MUD exhibited differences in the relative abundance of several microbial taxa. Importantly, the order *Lactobacillales*, containing several anti-inflammatory bacterial species, decreased in MUD and correlated positively with the plasma level of anti-inflammatory cytokine IL-10, as well as the duration of METH abstinence, suggesting a potential role of the gut-immune-brain axis (Fung et al., 2017) in MUD.

In our study, no difference was observed between the MUD and HC groups regarding the alpha diversity of fecal microbiota, which is in agreement with previously published literature (Cook et al., 2019). With respect to beta diversity, recent studies reported a significant variation in overall microbial composition between METH users and non-users (Fulcher et al., 2018; Cook et al., 2019), which was not replicated in the present study. This inconsistency may be due to the longer duration of abstinence in our study population. The METH-users in the aforementioned study reported METH use within 6 months (Cook et al., 2019), whereas the median duration of METH withdrawal in our study was 8.22 months. A recent study conducted in rats found that the significant difference in beta diversity caused by METH exposure was no longer evident after 7 days of cessation (Forouzan et al., 2021). Therefore, the similarity of the overall microbial structure between the MUD and HC groups in the present study might be explained by the restoration during prolonged abstinence.

According to our results, *Collinsella*, a genus belonging to the family *Coriobacteriaceae* and the phylum *Actinobacteria*, was recognized as the most robustly associated with MUD and positively related to the total years of METH use. In humans, *Collinsella* has been linked to poor metabolic states including obesity (Gomez-Arango et al., 2016; Frost et al., 2019), type 2 diabetes mellitus (Candela et al., 2016), hypercholesterolemia (Khan et al., 2018) and nonalcoholic steatohepatitis (Astbury et al., 2020). Additionally, *Collinsella* was considered as a biomarker in patients with symptomatic atherosclerosis (Karlsson et al., 2012). Since METH use is closely related to enhanced atherosclerotic plaque formation (Kevil et al., 2019), enrichment of *Collinsella* in MUD may indicate an increase susceptibility for cardiovascular disease, which is among the leading causes of death associated with MUD (Paulus and Stewart, 2020). Higher abundance of *Collinsella* has also been observed in patients with schizophrenia (Shen et al., 2018; Li et al., 2020). Given the considerable overlap in the clinical symptoms of METH-induced psychosis and schizophrenia (Wearne and Cornish, 2018), as well as the usage of METH in inducing animal models of schizophrenia-like phenotype (Machiyama, 1992; Oka et al., 2020), it is therefore likely to be a connection between *Collinsella* and psychotic symptoms in MUD, which was not investigated in this study. Furthermore, *Collinsella* has been associated with negative affect assessed by the Positive Affect and Negative Affect Schedule (PANAS) in healthy adults (Lee et al., 2020). However, no evidence of correlations between *Collinsella* and depression or anxiety was detected in the current study, possibly attributed to the differences in the scales used for measurement and the study population. With respect to substance use, Fulcher et al. (2018) found that decreased abundance of *Collinsella* was associated METH use during chronic HIV-infection among young men who have sex with men, which is inconsistent with our results. As participants with evidence of HIV infection were ruled out in our study, this contradiction may be due to the influence of uncontrolled confounders in Fulcher et al.’s study. Besides, a reduction of *Collinsella* has been indicated in alcohol use disorder (Litwinowicz et al., 2020). Notably, our results supported the negative association between *Collinsella* and alcohol use by incorporating the AUDIT score as a covariate (**Supplementary Table 1**). More importantly, *Collinsella* has been shown to influence the epithelial production of IL-17A and contribute to hyper-permeability of the gut by reducing the expression of the tight junction protein ZO-1 in research of rheumatoid arthritis (Chen et al., 2016). According to these data, we may infer that the observed higher abundance of *Collinsella* in MUD might further contribute to raise the host inflammatory level although no correlations between *Collinsella* and levels of inflammatory factors were found in our study.

In the current study, MUD was characterized by a reduction in the relative abundance of genera *Faecalibacterium*, *Blautia*, *Dorea* and *Streptococcus*, most of which are in line with previously reported data (Xu et al., 2017; Cook et al., 2019), although these associations did not remain significant after adjusting for confounders. Decreased abundance of *Faecalibacterium* has also been found in METH-users from a large cohort study (Cook et al., 2019). *Faecalibacterium prausnitzii*, the only identified species in the genus *Faecalibacterium*, is one of the most abundant butyrate-producing bacteria in the gastrointestinal tract and has demonstrated anti-inflammatory effects *via* NF-κB inhibition and IL-10 induction (Miquel et al., 2013). Thus, it is probable that the reduction in the abundance of *Faecalibacterium* may exacerbate inflammation in MUD. In contrast to our results, mice exposed to escalating dose-multiple binge METH presented a significantly higher abundance of *Blautia* (Chen et al., 2021), probably because of the species differences between human and rodent model. *Blautia* has been shown to play an important role in maintaining environmental balance in the intestine and preventing inflammation by upregulating intestinal regulatory T cells and producing short chain fatty acids (Kim et al., 2014). Nonetheless, it remains unclear whether *Blautia* is good for human health given that higher abundance of *Blautia* was detected under unhealthy conditions such as irritable bowel syndrome (Rajilić–Stojanović et al., 2011) and ulcerative colitis (Nishino et al., 2018) as well. In fact, it has been supposed that different species of *Blautia* may exert beneficial or adverse effects (Liu et al., 2021). In our study, *Blautia* was positively correlated with the plasma level of pro-inflammatory cytokine IL-2. Although the simultaneous increase of the relative abundance of *Blautia* and the level of IL-2 has been reported (Qi et al., 2019), further investigations are required to identify the relationship between *Blautia* and IL-2. In accord with our findings, a study comparing subjects with substance use disorder (SUD) with healthy controls reported a depletion of the genus *Dorea*, the genus *Streptococcus* and upper-level taxa including the family *Streptococcaceae*, the order *Lactobacillales* and the class *Bacilli* in the SUD group (Xu et al., 2017). Enrichment of the genus *Megasphaera* in the HC group was also reported (Xu et al., 2017); however, our study found the opposite. The discrepancy might be explained by the fact that participants with MUD made up only for 30% of the SUD group in the study.

Perhaps the most notable finding is that the order *Lactobacillales*, exhibiting lower abundance in the MUD group, was positively correlated with the duration of METH abstinence and the plasma level of anti-inflammatory cytokine IL-10. *Lactobacillales*, a dominant order in class *Bacilli* and phylum *Firmicutes*, is considered as a beneficial commensal. Bacteria in the order *Lactobacillales* produce lactic acid as a major end-product of carbohydrate fermentation and are among the most common microorganisms used as probiotics (Gareau et al., 2010; Lahtinen et al., 2011). Correlation analysis in our study indicated that the relative abundance of *Lactobacillales* increased as the withdrawal period progressed, suggesting a tendency towards restoration during abstinence. Bacterial strains belonging to *Lactobacillales* are known to have potential to modulate cytokine release (Gareau et al., 2010; Vemuri et al., 2017). Specifically, administration of *Lactobacillus casei* suppresses proinflammatory responses by increasing IL-10 levels (So et al., 2008b; So et al., 2008a). Interestingly, our results also revealed that *Lactobacillales* was positively correlated with IL-10 levels. While the mechanism for MUD-associated decrease in *Lactobacillales* remains unclear, the correlation between *Lactobacillales* and IL-10 would seem to suggest a potential role of the altered gut microbiota in MUD by modulating the systemic immune system of the host.

Though not the primary object of our study, it is somehow surprising to find that the frequency of METH use was conversely correlated with plasma levels of IL-6. In contrast, Luo et al. (2021) found a positive association between IL-6 and frequency of METH use among chronic METH users with an average abstinence time of about 40 days, much shorter than that of our subjects. Another study in which alcohol-dependent subjects hospitalized for a 3-week detoxification program reported a positive correlation between IL-10 and alcohol craving at onset of withdrawal (Leclercq et al., 2012). After 3 weeks of abstinence, however, IL-10 was found negatively relating to depression, anxiety and craving (Leclercq et al., 2012). As in our study, IL-10 was positively correlated with craving for METH while negatively correlated with anxiety measured by GAD-7. In addition, CRP, a typical inflammation marker, presented negative correlations with the frequency of METH use and craving in our study. Many of findings were contrary to expectations that higher pro-inflammatory markers would relate to more frequent METH use, stronger craving and more severe negative affect (i.e., depression and anxiety) while higher anti-inflammatory cytokines would relate to less frequent METH use, lower craving and less severe negative affect. These rather disturbing results might indicate a more complex relationship between immune system activity and addiction as mentioned by (Stamatovich et al., 2021) in a previous study of cocaine use disorder, possibly distinct across different periods of substance use.

Several limitations of this study need to be addressed. First, the present work should be interpreted as exploratory owing to the relatively small sample size consisting of male subjects only and the imbalance of the size between the two groups. Besides, the cross-sectional design of our study does not allow for conclusions about the causality of the demonstrated correlations. Thus, prospective research with a larger cohort is warranted. Further studies involving *in vitro* and *in vivo* host-microbe interaction are also needed to understand causal mechanisms. Second, plasma levels of systemic inflammatory factors and the severity of negative affect were not measured in the HC group; thus, whether these indicators were abnormal in the MUD group remains unclear in this study. Although higher levels of depression and anxiety in METH users, as well as the METH-associated changes in inflammatory factors have been reported (Huckans et al., 2015; Papageorgiou et al., 2019), results concerning these metrics in our study should be interpreted cautiously. Third, despite the fact that this study was controlled for factors affecting the gut microbiota, including age, BMI, alcohol use and smoking, other confounding factors such as diet, physical activity and socio-economic variables such as economic status and level of education should be fully considered in future studies. Fourth, this study was limited to a panel of only five markers of systemic inflammation. A greater scope of cytokines, chemokines and other molecular factors related to METH use (e.g., IL-1β, IL-8, IL-12, IL-18, interferon-gamma and Intercellular adhesion molecule 1; Papageorgiou et al., 2019) should be evaluated in further investigations. Moreover, our study included only a brief battery of self-reported questionnaires. Although we did find significant correlations between fecal microbiota, immune factors and clinical variables, future studies could apply a more comprehensive assessment involving cognitive function and psychotic symptoms, which is also an issue of concern. Fifth, the current study used only VAS as a subjective evaluation to assess craving for METH, which may lead to reporting bias and social desirability bias, especially among subjects undergoing compulsory rehabilitation in this study. Although VAS has been commonly used for craving assessment in multiple studies (Rosenberg, 2009; Kleykamp et al., 2019), future research could apply objective methods (e.g., electroencephalograph) to improve the reliability of craving measurement (Parvaz et al., 2016). Finally, the use of 16S rRNA sequencing only allowed us to identify taxa down to the genus level, and specific variation at the species level was therefore not evaluated. It is now generally accepted that different bacterial species, or even strains, of the same genus may exert completely different effects on the host (Mihatsch et al., 2012). Therefore, metagenomics studies are necessary to pursue in-depth understanding in this regard.

In summary, our findings provide evidence for alterations of fecal microbiota in male subjects with MUD. Furthermore, we demonstrated, for the first time, that altered microbial taxa were correlated with the levels of specific systemic inflammatory factors and clinical characteristics in MUD. As inflammation is one of the proposed mechanisms through which gut microbiota could potentially regulate aberrant brain functions in MUD, further revealing the precise interactions between gut microbiota and systemic inflammation may provide mechanistic insights and help to develop new therapeutic approaches for individuals with MUD.

## Data Availability Statement

The data presented in the study are deposited in the National Center for Biotechnology Information (NCBI) BioProject database with project number PRJNA766176. http://www.ncbi.nlm.nih.gov/bioproject/766176.

## Ethics Statement

The studies involving human participants were reviewed and approved by the Institutional Review Board and the Ethics Committee of Shanghai Mental Health Center. The patients/participants provided their written informed consent to participate in this study.

## Author Contributions

Conceptualization, DD and MZ. Data curation, DD, HS, YS, TC, and QS. Formal analysis, DD. Funding acquisition, MZ. Investigation, DD, HS, YS, and TC. Project administration, QS. Supervision, HJ and MZ. Writing—original draft, DD. Writing—review & editing, DD and MZ. All authors contributed to the article and approved the submitted version.

## Funding

This work was supported by the National Nature Science Foundation of China (81771436, 82130041), Shanghai Municipal Science and Technology Major Project (2018SHZDZX05), Shanghai Shenkang Hospital Development Center (SHDC2020CR3045B), Shanghai Clinical Research Center for Mental Health (19MC1911100), Shanghai Engineering Research Center of Intelligent Addiction Treatment and Rehabilitation (19DZ2255200) and Shanghai Key Laboratory of Psychotic Disorders (13DZ2260500).

## Conflict of Interest

The authors declare that the research was conducted in the absence of any commercial or financial relationships that could be construed as a potential conflict of interest.

## Publisher’s Note

All claims expressed in this article are solely those of the authors and do not necessarily represent those of their affiliated organizations, or those of the publisher, the editors and the reviewers. Any product that may be evaluated in this article, or claim that may be made by its manufacturer, is not guaranteed or endorsed by the publisher.

## References

[B1] Agirman G. Hsiao E. Y. (2021). SnapShot: The Microbiota-Gut-Brain Axis. Cell 184, 2524–2524.e1. doi: 10.1016/j.cell.2021.03.022 33930299

[B2] Aho V. T. E. Pereira P. A. B. Voutilainen S. Paulin L. Pekkonen E. Auvinen P.. (2019). Gut Microbiota in Parkinson’s Disease: Temporal Stability and Relations to Disease Progression. EBioMedicine 44, 691–707. doi: 10.1016/j.ebiom.2019.05.064 31221587 PMC6606744

[B3] Ames N. J. Barb J. J. Schuebel K. Mudra S. Meeks B. K. Tuason R. T. S.. (2020). Longitudinal Gut Microbiome Changes in Alcohol Use Disorder are Influenced by Abstinence and Drinking Quantity. Gut Microbes 11, 1608–1631. doi: 10.1080/19490976.2020.1758010 32615913 PMC7527072

[B4] Angoa-Pérez M. Zagorac B. Winters A. D. Greenberg J. M. Ahmad M. Theis K. R.. (2020). Differential Effects of Synthetic Psychoactive Cathinones and Amphetamine Stimulants on the Gut Microbiome in Mice. PLoS One 15, 1–18. doi: 10.1371/journal.pone.0227774 PMC6980639 31978078

[B5] Astbury S. Atallah E. Vijay A. Aithal G. P. Grove J. I. Valdes A. M. (2020). Lower Gut Microbiome Diversity and Higher Abundance of Proinflammatory Genus Collinsella are Associated With Biopsy-Proven Nonalcoholic Steatohepatitis. Gut Microbes 11, 569–580. doi: 10.1080/19490976.2019.1681861 31696774 PMC7524262

[B6] Barr T. Helms C. Grant K. Messaoudi I. (2016). Opposing Effects of Alcohol on the Immune System. Prog. Neuropsychopharmacol. Biol. Psychiatry 65, 242–251. doi: 10.1016/j.pnpbp.2015.09.001 26375241 PMC4911891

[B7] Bastiaanssen T. F. S. Cussotto S. Claesson M. J. Clarke G. Dinan T. G. Cryan J. F. (2020). Gutted! Unraveling the Role of the Microbiome in Major Depressive Disorder. Harv. Rev. Psychiatry 28, 26–39. doi: 10.1097/HRP.0000000000000243 31913980 PMC7012351

[B8] Bulgart H. R. Neczypor E. W. Wold L. E. Mackos A. R. (2020). Microbial Involvement in Alzheimer Disease Development and Progression. Mol. Neurodegener. 15, 1–12. doi: 10.1186/s13024-020-00378-4 32709243 PMC7382139

[B9] Candela M. Biagi E. Soverini M. Consolandi C. Quercia S. Severgnini M.. (2016). Modulation of Gut Microbiota Dysbioses in Type 2 Diabetic Patients by Macrobiotic Ma-Pi 2 Diet. Br. J. Nutr. 116, 80–93. doi: 10.1017/S0007114516001045 27151248 PMC4894062

[B10] Caporaso J. G. Kuczynski J. Stombaugh J. Bittinger K. Bushman F. D. Costello E. K.. (2010). Correspondence QIIME Allows Analysis of High- Throughput Community Sequencing Data Intensity Normalization Improves Color Calling in SOLiD Sequencing. Nat. Publ. Gr. 7, 335–336. doi: 10.1038/nmeth0510-335 PMC3156573 20383131

[B11] Chen J. Wright K. Davis J. M. Jeraldo P. Marietta E. V. Murray J.. (2016). An Expansion of Rare Lineage Intestinal Microbes Characterizes Rheumatoid Arthritis. Genome Med. 8, 1–14. doi: 10.1186/s13073-016-0299-7 27102666 PMC4840970

[B12] Chen L. J. Zhi X. Zhang K. K. Wang L. Li J. H. Liu J. L.. (2021). Escalating Dose-Multiple Binge Methamphetamine Treatment Elicits Neurotoxicity, Altering Gut Microbiota and Fecal Metabolites in Mice. Food Chem. Toxicol. 148:111946. doi: 10.1016/j.fct.2020.111946 33359793

[B13] Cole J. R. Wang Q. Fish J. A. Chai B. McGarrell D. M. Sun Y.. (2014). Ribosomal Database Project: Data and Tools for High Throughput rRNA Analysis. Nucleic Acids Res. 42, 633–642. doi: 10.1093/nar/gkt1244 PMC3965039 24288368

[B14] Cook R. R. Fulcher J. A. Tobin N. H. Li F. Lee D. J. Woodward C.. (2019). Alterations to the Gastrointestinal Microbiome Associated With Methamphetamine Use Among Young Men Who Have Sex With Men. Sci. Rep. 9, 1–11. doi: 10.1038/s41598-019-51142-8 31619731 PMC6795845

[B15] Cryan J. F. O’Riordan K. J. Cowan C. S. M. Sandhu K. V. Bastiaanssen T. F. S. Boehme M.. (2019). The Microbiota-Gut-Brain Axis. Physiol. Rev. 99, 1877–2013. doi: 10.1152/physrev.00018.2018 31460832

[B16] Doggui R. Elsawy W. Conti A. A. Baldacchino A. (2021). Association Between Chronic Psychoactive Substances Use and Systemic Inflammation: A Systematic Review and Meta-Analysis. Neurosci. Biobehav. Rev. 125, 208–220. doi: 10.1016/j.neubiorev.2021.02.031 33639179

[B17] Edgar R. C. (2013). UPARSE: Highly Accurate OTU Sequences From Microbial Amplicon Reads. Nat. Methods 10, 996–998. doi: 10.1038/nmeth.2604 23955772

[B18] Forouzan S. Hoffman K. L. Kosten T. A. (2021). Methamphetamine Exposure and its Cessation Alter Gut Microbiota and Induce Depressive-Like Behavioral Effects on Rats. Psychopharmacol. (Berl). 238, 281–292. doi: 10.1007/s00213-020-05681-y 33097978

[B19] Frost F. Storck L. J. Kacprowski T. Gärtner S. Rühlemann M. Bang C.. (2019). A Structured Weight Loss Program Increases Gut Microbiota Phylogenetic Diversity and Reduces Levels of Collinsella in Obese Type 2 Diabetics: A Pilot Study. PLoS One 14, 1–14. doi: 10.1371/journal.pone.0219489 PMC6638920 31318902

[B20] Fulcher J. A. Hussain S. K. Cook R. Li F. Tobin N. H. Ragsdale A.. (2018). Effects of Substance Use and Sex Practices on the Intestinal Microbiome During HIV-1 Infection. J. Infect. Dis. 218, 1560–1570. doi: 10.1093/infdis/jiy349 29982500 PMC6692862

[B21] Fung T. C. Olson C. A. Hsiao E. Y. (2017). Interactions Between the Microbiota, Immune and Nervous Systems in Health and Disease. Nat. Neurosci. 20, 145–155. doi: 10.1038/nn.4476 28092661 PMC6960010

[B22] Gareau M. G. Sherman P. M. Walker W. A. (2010). Probiotics and the Gut Microbiota in Intestinal Health and Disease. Nat. Rev. Gastroenterol. Hepatol. 7, 503–514. doi: 10.1038/nrgastro.2010.117 20664519 PMC4748966

[B23] Golofast B. Vales K. (2020). The Connection Between Microbiome and Schizophrenia. Neurosci. Biobehav. Rev. 108, 712–731. doi: 10.1016/j.neubiorev.2019.12.011 31821833

[B24] Gomez-Arango L. F. Barrett H. L. McIntyre H. D. Callaway L. K. Morrison M. Nitert M. D.. (2016). Connections Between the Gut Microbiome and Metabolic Hormones in Early Pregnancy in Overweight and Obese Women. Diabetes 65, 2214–2223. doi: 10.2337/db16-0278 27217482

[B25] Gu Z. Gu L. Eils R. Schlesner M. Brors B. (2014). Circlize Implements and Enhances Circular Visualization in R. Bioinformatics 30, 2811–2812. doi: 10.1093/bioinformatics/btu393 24930139

[B26] Heatherton T. F. Kozlowski L. T. Frecker R. C. Fagerstrom K.-O. (1991). The Fagerström Test for Nicotine Dependence: A Revision of the Fagerstrom Tolerance Questionnaire. Br. J. Addict. 86, 1119–1127. doi: 10.1111/j.1360-0443.1991.tb01879.x 1932883

[B27] Huckans M. Fuller B. E. Chalker A. L. N. Adams M. Loftis J. M. (2015). Plasma Inflammatory Factors are Associated With Anxiety, Depression, and Cognitive Problems in Adults With and Without Methamphetamine Dependence: An Exploratory Protein Array Study. Front. Psychiatry 6:178. doi: 10.3389/fpsyt.2015.00178 26732994 PMC4683192

[B28] Jiang H. Zhang X. Yu Z. Zhang Z. Deng M. Zhao J.H.. (2018). Altered Gut Microbiota Profile in Patients With Generalized Anxiety Disorder. J. Psychiatr. Res. 104, 130–136. doi: 10.1016/j.jpsychires.2018.07.007 30029052

[B29] Kanehisa M. Sato Y. Kawashima M. Furumichi M. Tanabe M. (2016). KEGG as a Reference Resource for Gene and Protein Annotation. Nucleic Acids Res. 44, D457–D462. doi: 10.1093/nar/gkv1070 26476454 PMC4702792

[B30] Karlsson F. H. Fåk F. Nookaew I. Tremaroli V. Fagerberg B. Petranovic D.. (2012). Symptomatic Atherosclerosis is Associated With an Altered Gut Metagenome. Nat. Commun. 3, 1245. doi: 10.1038/ncomms2266 23212374 PMC3538954

[B31] Kevil C. G. Goeders N. E. Woolard M. D. Bhuiyan M. S. Dominic P. Kolluru G. K.. (2019). Methamphetamine Use and Cardiovascular Disease. Arterioscler. Thromb. Vasc. Biol. 39, 1739–1746. doi: 10.1161/ATVBAHA.119.312461 31433698 PMC6709697

[B32] Khan T. J. Ahmed Y. M. Zamzami M. A. Siddiqui A. M. Khan I. Baothman O. A. S.. (2018). Atorvastatin Treatment Modulates the Gut Microbiota of the Hypercholesterolemic Patients. Omi. A J. Integr. Biol. 22, 154–163. doi: 10.1089/omi.2017.0130 29432061

[B33] Kim C. H. Park J. Kim M. (2014). Gut Microbiota-Derived Short-Chain Fatty Acids, T Cells, and Inflammation. Immune Netw. 14, 277. doi: 10.4110/in.2014.14.6.277 25550694 PMC4275385

[B34] Kleykamp B. A. De Santis M. Dworkin R. H. Huhn A. S. Kampman K. M. Montoya I. D.. (2019). Craving and Opioid Use Disorder: A Scoping Review. Drug Alcohol Depend. 205, 107639. doi: 10.1016/j.drugalcdep.2019.107639 31683241

[B35] Kroenke K. Spitzer R. L. Williams J. B. (2001). The PHQ-9: Validity of a Brief Depression Severity Measure. J. Gen. Intern. Med. 16, 606–613. doi: 10.1046/j.1525-1497.2001.016009606.x 11556941 PMC1495268

[B36] Lahtinen S. Ouwehand A. C. Salminen S. von Wright A. (2011). Lactic Acid Bacteria: Microbiological and Functional Aspects, 4th ed. Boca Raton: CRC Press. doi: 10.3920/bm2012.x003

[B37] Langille M. G. I. Zaneveld J. Caporaso J. G. McDonald D. Knights D. Reyes J. A.. (2013). Predictive Functional Profiling of Microbial Communities Using 16S rRNA Marker Gene Sequences. Nat. Biotechnol. 31, 814–821. doi: 10.1038/nbt.2676 23975157 PMC3819121

[B38] Leclercq S. Cani P. D. Neyrinck A. M. Stärkel P. Jamar F. Mikolajczak M.. (2012). Role of Intestinal Permeability and Inflammation in the Biological and Behavioral Control of Alcohol-Dependent Subjects. Brain. Behav. Immun. 26, 911–918. doi: 10.1016/j.bbi.2012.04.001 22521198

[B39] Leclercq S. Matamoros S. Cani P. D. Neyrinck A. M. Jamar F. Stärkel P.. (2014). Intestinal Permeability, Gut-Bacterial Dysbiosis, and Behavioral Markers of Alcohol-Dependence Severity. Proc. Natl. Acad. Sci. U. S. A. 111, E4485–E4493. doi: 10.1073/pnas.1415174111 25288760 PMC4210345

[B40] Lee S. H. Yoon S. H. Jung Y. Kim N. Min U. Chun J.. (2020). Emotional Well-Being and Gut Microbiome Profiles by Enterotype. Sci. Rep. 10, 1–9. doi: 10.1038/s41598-020-77673-z 33244049 PMC7691370

[B41] Leung M. H. Y. Tong X. Bastien P. Guinot F. Tenenhaus A. Appenzeller B. M. R.. (2020). Changes of the Human Skin Microbiota Upon Chronic Exposure to Polycyclic Aromatic Hydrocarbon Pollutants. Microbiome 8, 1–17. doi: 10.1186/s40168-020-00874-1 32591010 PMC7320578

[B42] Lim M. Y. Yoon H. S. Rho M. Sung J. Song Y. M. Lee K.. (2016). Analysis of the Association Between Host Genetics, Smoking, and Sputum Microbiota in Healthy Humans. Sci. Rep. 6, 1–9. doi: 10.1038/srep23745 27030383 PMC4814871

[B43] Litwinowicz K. Choroszy M. Waszczuk E. (2020). Changes in the Composition of the Human Intestinal Microbiome in Alcohol Use Disorder: A Systematic Review. Am. J. Drug Alcohol Abuse 46, 4–12. doi: 10.1080/00952990.2019.1669629 31689142

[B44] Liu X. Mao B. Gu J. Wu J. Cui S. Wang G.. (2021). Blautia—a New Functional Genus With Potential Probiotic Properties? Gut Microbes 13, 1–21. doi: 10.1080/19490976.2021.1875796 PMC7872077 33525961

[B45] Li S. Zhuo M. Huang X. Huang Y. Zhou J. Xiong D.. (2020). Altered Gut Microbiota Associated With Symptom Severity in Schizophrenia. PeerJ 8, 1–22. doi: 10.7717/peerj.9574 PMC7395597 32821537

[B46] London E. D. Simon S. L. Berman S. M. Mandelkern M. A. Lichtman A. M. Bramen J.. (2004). Mood Disturbances and Regional Cerebral Metabolic Abnormalities in Recently Abstinent Methamphetamine Abusers. Arch. Gen. Psychiatry 61, 73–84. doi: 10.1001/archpsyc.61.1.73 14706946

[B47] Lozupone C. A. Hamady M. Kelley S. T. Knight R. (2007). Quantitative and Qualitative β Diversity Measures Lead to Different Insights Into Factors That Structure Microbial Communities. Appl. Environ. Microbiol. 73, 1576–1585. doi: 10.1128/AEM.01996-06 17220268 PMC1828774

[B48] Lozupone C. Knight R. (2005). UniFrac: A New Phylogenetic Method for Comparing Microbial Communities. Appl. Environ. Microbiol. 71, 8228–8235. doi: 10.1128/AEM.71.12.8228-8235.2005 16332807 PMC1317376

[B49] Luo Y. He H. Ou Y. Zhou Y. Fan N. (2021). Elevated Serum Levels of TNF-α, IL-6, and IL-18 in Chronic Methamphetamine Users. Hum. Psychopharmacol. Clin. Exp. e2810, 1–8. doi: 10.1002/hup.2810 34432333

[B50] Machiyama Y. (1992). Chronic Methamphetamine Intoxication Model of Schizophrenia in Animals. Schizophr. Bull. 18, 107–113. doi: 10.1093/schbul/18.1.107 1553490

[B51] Mallick H. Rahnavard A. McIver L. J. Ma S. Zhang Y. Nguyen L. H.. (2021). Multivariable Association Discovery in Population-Scale Meta-Omics Studies. bioRxiv. 2021.01.20.427420. doi: 10.1101/2021.01.20.427420 PMC8714082 34784344

[B52] Masella A. P. Bartram A. K. Truszkowski J. M. Brown D. G. Neufeld J. D. (2012). PANDAseq: Paired-End Assembler for Illumina Sequences. BMC Bioinf. 13:31. doi: 10.1186/1471-2105-13-31 PMC3471323 22333067

[B53] Mihatsch W. A. Braegger C. P. Decsi T. Kolacek S. Lanzinger H. Mayer B.. (2012). Critical Systematic Review of the Level of Evidence for Routine Use of Probiotics for Reduction of Mortality and Prevention of Necrotizing Enterocolitis and Sepsis in Preterm Infants. Clin. Nutr. 31, 6–15. doi: 10.1016/j.clnu.2011.09.004 21996513

[B54] Miquel S. Martín R. Rossi O. Bermúdez-Humarán L. G. Chatel J. M. Sokol H.. (2013). Faecalibacterium Prausnitzii and Human Intestinal Health. Curr. Opin. Microbiol. 16, 255–261. doi: 10.1016/j.mib.2013.06.003 23831042

[B55] Ning T. Gong X. Xie L. Ma B. (2017). Gut Microbiota Analysis in Rats With Methamphetamine-Induced Conditioned Place Preference. Front. Microbiol. 8:1620. doi: 10.3389/fmicb.2017.01620 28890714 PMC5575146

[B56] Nishino K. Nishida A. Inoue R. Kawada Y. Ohno M. Sakai S.. (2018). Analysis of Endoscopic Brush Samples Identified Mucosa-Associated Dysbiosis in Inflammatory Bowel Disease. J. Gastroenterol. 53, 95–106. doi: 10.1007/s00535-017-1384-4 28852861

[B57] Noguera-Julian M. Rocafort M. Guillén Y. Rivera J. Casadellà M. Nowak P.. (2016). Gut Microbiota Linked to Sexual Preference and HIV Infection. EBioMedicine 5, 135–146. doi: 10.1016/j.ebiom.2016.01.032 27077120 PMC4816837

[B58] Office of China National Narcotics Control Commission (2020) Drug Situation in China (2019). Available at: http://www.nncc626.com/2020-06/25/c_1210675877.htm (Accessed September 8, 2021).

[B59] Oka M. Ito K. Koga M. Kusumi I. (2020). Changes in Subunit Composition of NMDA Receptors in Animal Models of Schizophrenia by Repeated Administration of Methamphetamine. Prog. Neuropsychopharmacol. Biol. Psychiatry 103:109984. doi: 10.1016/j.pnpbp.2020.109984 32473191

[B60] Papageorgiou M. Raza A. Fraser S. Nurgali K. Apostolopoulos V. (2019). Methamphetamine and its Immune-Modulating Effects. Maturitas 121, 13–21. doi: 10.1016/j.maturitas.2018.12.003 30704560

[B61] Parvaz M. A. Moeller S. J. Goldstein R. Z. (2016). Incubation of Cue-Induced Craving in Adults Addicted to Cocaine Measured by Electroencephalography. JAMA Psychiatry 73, 1127–1134. doi: 10.1001/jamapsychiatry.2016.2181 27603142 PMC5206796

[B62] Paulus M. P. Stewart J. L. (2020). Neurobiology, Clinical Presentation, and Treatment of Methamphetamine Use Disorder: A Review. JAMA Psychiatry 77, 959–966. doi: 10.1001/jamapsychiatry.2020.0246 32267484 PMC8098650

[B63] Petrov V. A. Saltykova I. V. Zhukova I. A. Alifirova V. M. Zhukova N. G. Dorofeeva Y. B.. (2017). Analysis of Gut Microbiota in Patients With Parkinson’s Disease. Bull. Exp. Biol. Med. 162, 734–737. doi: 10.1007/s10517-017-3700-7 28429209

[B64] Prakash M. D. Tangalakis K. Antonipillai J. Stojanovska L. Nurgali K. Apostolopoulos V. (2017). Methamphetamine: Effects on the Brain, Gut and Immune System. Pharmacol. Res. 120, 60–67. doi: 10.1016/j.phrs.2017.03.009 28302577

[B65] Qi Y. Chen L. Gao K. Shao Z. Huo X. Hua M.. (2019). Effects of Schisandra Chinensis Polysaccharides on Rats With Antibiotic-Associated Diarrhea. Int. J. Biol. Macromol. 124, 627–634. doi: 10.1016/j.ijbiomac.2018.11.250 30500495

[B66] Qin C. Hu J. Wan Y. Cai M. Wang Z. Peng Z.. (2021). Narrative Review on Potential Role of Gut Microbiota in Certain Substance Addiction. Prog. Neuropsychopharmacol. Biol. Psychiatry 106, 110093. doi: 10.1016/j.pnpbp.2020.110093 32898589

[B67] Rajilić–Stojanović M. Biagi E. Heilig H. G. H. J. Kajander K. Kekkonen R. A. Tims S.. (2011). Global and Deep Molecular Analysis of Microbiota Signatures in Fecal Samples From Patients With Irritable Bowel Syndrome. Gastroenterology 141, 1792–1801. doi: 10.1053/j.gastro.2011.07.043 21820992

[B68] R Core Team. (2021). R: A Language and Environment for Statistical Computing (Vienna, Austria: R Foundation for Statistical Computing). Available at: https://www.R-project.org/.

[B69] Rommel N. Rohleder N. H. Wagenpfeil S. Haertel-Petri R. Kesting M. R. (2015). Evaluation of Methamphetamine-Associated Socioeconomic Status and Addictive Behaviors, and Their Impact on Oral Health. Addict. Behav. 50, 182–187. doi: 10.1016/j.addbeh.2015.06.040 26151583

[B70] Rosenberg H. (2009). Clinical and Laboratory Assessment of the Subjective Experience of Drug Craving. Clin. Psychol. Rev. 29, 519–534. doi: 10.1016/j.cpr.2009.06.002 19577831

[B71] Salavrakos M. Leclercq S. De Timary P. Dom G. (2021). Microbiome and Substances of Abuse. Prog. Neuropsychopharmacol. Biol. Psychiatry 105:110113. doi: 10.1016/j.pnpbp.2020.110113 32971216

[B72] Saunders J. B. Aasland O. G. Babor T. F. de la Fuente J. R. Grant M. (1993). Development of the Alcohol Use Disorders Identification Test (AUDIT): WHO Collaborative Project on Early Detection of Persons With Harmful Alcohol Consumption-II. Addiction 88, 791–804. doi: 10.1111/j.1360-0443.1993.tb02093.x 8329970

[B73] Segata N. Izard J. Waldron L. Gevers D. Miropolsky L. Garrett W. S.. (2011). Metagenomic Biomarker Discovery and Explanation. Genome Biol. 12, R60. doi: 10.1186/gb-2011-12-6-r60 21702898 PMC3218848

[B74] Sharon G. Cruz N. J. Kang D. W. Gandal M. J. Wang B. Kim Y. M.. (2019). Human Gut Microbiota From Autism Spectrum Disorder Promote Behavioral Symptoms in Mice. Cell 177, 1600–1618.e17. doi: 10.1016/j.cell.2019.05.004 31150625 PMC6993574

[B75] Shen Y. Xu J. Li Z. Huang Y. Yuan Y. Wang J.. (2018). Analysis of Gut Microbiota Diversity and Auxiliary Diagnosis as a Biomarker in Patients With Schizophrenia: A Cross-Sectional Study. Schizophr. Res. 197, 470–477. doi: 10.1016/j.schres.2018.01.002 29352709

[B76] So J. S. Lee C. G. Kwon H. K. Yi H. J. Chae C. S. Park J. A.. (2008b). Lactobacillus Casei Potentiates Induction of Oral Tolerance in Experimental Arthritis. Mol. Immunol. 46, 172–180. doi: 10.1016/j.molimm.2008.07.038 18804867

[B77] So J. S. Kwon H. K. Lee C. G. Yi H. J. Park J. A. Lim S. Y.. (2008a). Lactobacillus Casei Suppresses Experimental Arthritis by Down-Regulating T Helper 1 Effector Functions. Mol. Immunol. 45, 2690–2699. doi: 10.1016/j.molimm.2007.12.010 18243320

[B78] Spitzer R. L. Kroenke K. Williams J. B. W. Löwe B. (2006). A Brief Measure for Assessing Generalized Anxiety Disorder: The GAD-7. Arch. Intern. Med. 166, 1092–1097. doi: 10.1001/archinte.166.10.1092 16717171

[B79] Stamatovich S. N. Lopez-Gamundi P. Suchting R. Colpo G. D. Walss-Bass C. Lane S. D.. (2021). Plasma Pro- and Anti-Inflammatory Cytokines may Relate to Cocaine Use, Cognitive Functioning, and Depressive Symptoms in Cocaine Use Disorder. Am. J. Drug Alcohol Abuse 47, 52–64. doi: 10.1080/00952990.2020.1828439 33119414

[B80] Sureshchandra S. Raus A. Jankeel A. Ligh B. J. K. Walter N. A. R. Newman N.. (2019). Dose-Dependent Effects of Chronic Alcohol Drinking on Peripheral Immune Responses. Sci. Rep. 9, 1–13. doi: 10.1038/s41598-019-44302-3 31127176 PMC6534547

[B81] United Nations Office on Drugs and Crime (2021) Booklet 4 - Drug Market Trends: Cocaine, Amphetamine-Type Stimulants. Available at: https://www.unodc.org/unodc/en/data-and-analysis/wdr-2021_booklet-4.html.

[B82] Vemuri R. Gundamaraju R. Eri R. (2017). Role of Lactic Acid Probiotic Bacteria in IBD. Curr. Pharm. Des. 23, 2352–2355. doi: 10.2174/1381612823666170207100025 28176664

[B83] Vogt N. M. Kerby R. L. Dill-McFarland K. A. Harding S. J. Merluzzi A. P. Johnson S. C.. (2017). Gut Microbiome Alterations in Alzheimer’s Disease. Sci. Rep. 7, 1–11. doi: 10.1038/s41598-017-13601-y 29051531 PMC5648830

[B84] Wang Q. Garrity G. M. Tiedje J. M. Cole J. R. (2007). Naïve Bayesian Classifier for Rapid Assignment of rRNA Sequences Into the New Bacterial Taxonomy. Appl. Environ. Microbiol. 73, 5261–5267. doi: 10.1128/AEM.00062-07 17586664 PMC1950982

[B85] Wang M. Wan J. Rong H. He F. Wang H. Zhou J.. (2019). Alterations in Gut Glutamate Metabolism Associated With Changes in Gut Microbiota Composition in Children With Autism Spectrum Disorder. mSystems 4, 61. doi: 10.1128/mSystems.00321-18 PMC6351726 30701194

[B86] Wang Y. Zhang J. Deji C. Fan J. Miao X. Li S.. (2021). Differential Perturbations of Gut Microbial Profiles and Co-Occurrence Networks Among Phases of Methamphetamine-Induced Conditioned Place Preference. J. Neurosci. Res. 00, 1–14. doi: 10.1002/jnr.24963 34510511

[B87] Wannamethee S. G. Lowe G. D. O. Shaper A. G. Rumley A. Lennon L. Whincup P. H. (2005). Associations Between Cigarette Smoking, Pipe/Cigar Smoking, and Smoking Cessation, and Haemostatic and Inflammatory Markers for Cardiovascular Disease. Eur. Heart J. 26, 1765–1773. doi: 10.1093/eurheartj/ehi183 15817606

[B88] Wearne T. A. Cornish J. L. (2018). A Comparison of Methamphetamine-Induced Psychosis and Schizophrenia: A Review of Positive, Negative, and Cognitive Symptomatology. Front. Psychiatry 9:491. doi: 10.3389/fpsyt.2018.00491 30364176 PMC6191498

[B89] Wickham H. (2016). Ggplot2: Elegant Graphics for Data Analysis (New York: Springer-Verlag).

[B90] Xu Y. Xie Z. Wang H. Shen Z. Guo Y. Gao Y.. (2017). Bacterial Diversity of Intestinal Microbiota in Patients With Substance Use Disorders Revealed by 16S rRNA Gene Deep Sequencing. Sci. Rep. 7, 1–9. doi: 10.1038/s41598-017-03706-9 28620208 PMC5472629

[B91] Yang C. Fu X. Hao W. Xiang X. Liu T. Yang B. Z.. (2021). Gut Dysbiosis Associated With the Rats’ Responses in Methamphetamine-Induced Conditioned Place Preference. Addict. Biol. 26, e12975. doi: 10.1111/adb.12975 33094505

[B92] Yang Z. Li J. Gui X. Shi X. Bao Z. Han H.. (2020). Updated Review of Research on the Gut Microbiota and Their Relation to Depression in Animals and Human Beings. Mol. Psychiatry 25, 2759–2772. doi: 10.1038/s41380-020-0729-1 32332994

[B93] Yang Y. Yu X. Liu X. Liu G. Zeng K. Wang G. (2021). Altered Fecal Microbiota Composition in Individuals Who Abuse Methamphetamine. Sci. Rep. 11, 1–13. doi: 10.1038/s41598-021-97548-1 34518605 PMC8437956

[B94] Zhu F. Guo R. Wang W. Ju Y. Wang Q. Ma Q.. (2019). Transplantation of Microbiota From Drug-Free Patients With Schizophrenia Causes Schizophrenia-Like Abnormal Behaviors and Dysregulated Kynurenine Metabolism in Mice. Mol. Psychiatry 25, 2905–2918 doi: 10.1038/s41380-019-0475 31391545

