# Supplementary material for: Altered Fecal Microbiota Correlated With Systemic Inflammation in Male Subjects With Methamphetamine Use Disorder
Source: Front Cell Infect Microbiol. 2021 Nov 18;11:783917. doi: 10.3389/fcimb.2021.783917 (PMC8637621; doi:10.3389/fcimb.2021.783917)
Supplement: Supplementary file 1 [file DataSheet_1.pdf]

Supplementary Figure 1. Enrollment of subjects.

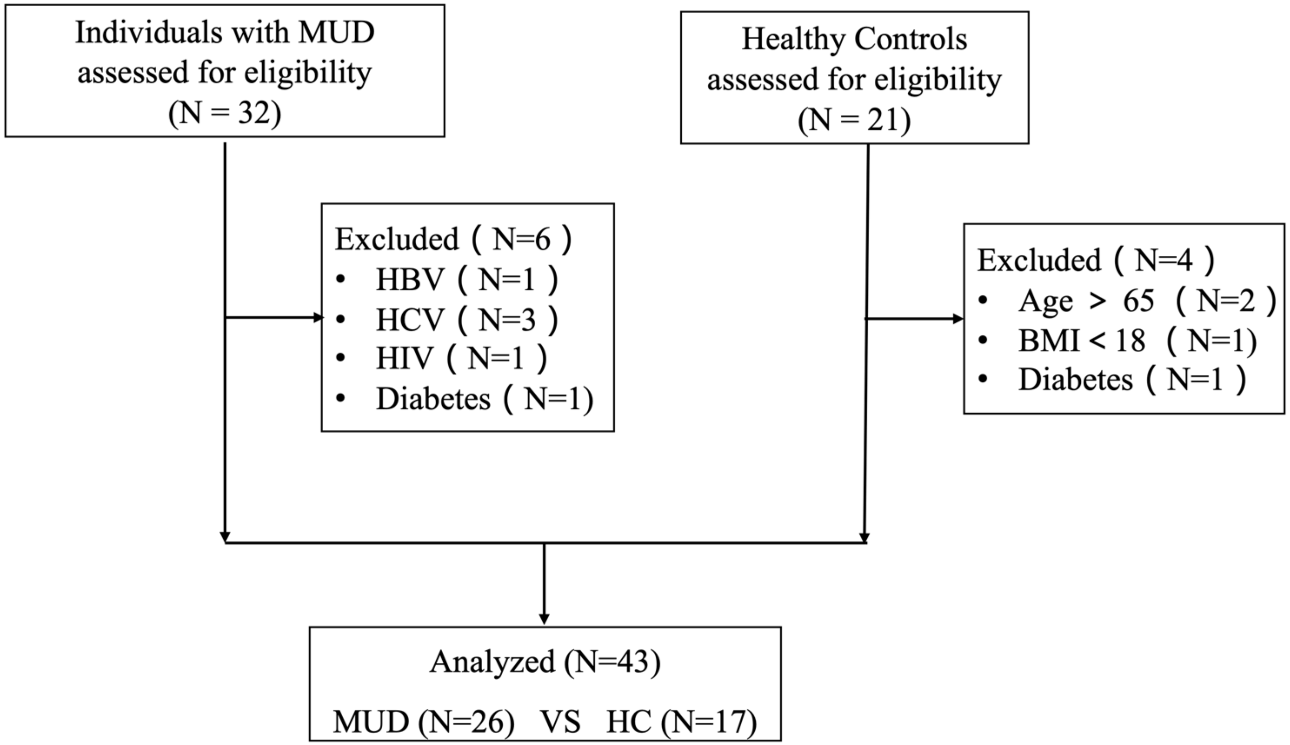

Supplementary Figure 2. OTU-level diversity and alpha diversity between the MUD and HC groups. **(A)** Venn diagram showing common and unique sets of OTUs between the groups. **(B)** Boxplots depicting α-diversity between the groups as measured by the Chao1, Observed Species, Shannon and Simpson indices. The lower and upper hinges and middle line of boxplots correspond to the 25th, 75th and 50th percentiles. Whiskers represented the most extreme data within 1.5 times of the interquartile range. Outliers beyond the whiskers are displayed as dots. Wilcoxon rank sum test, all $p > 0.05$.

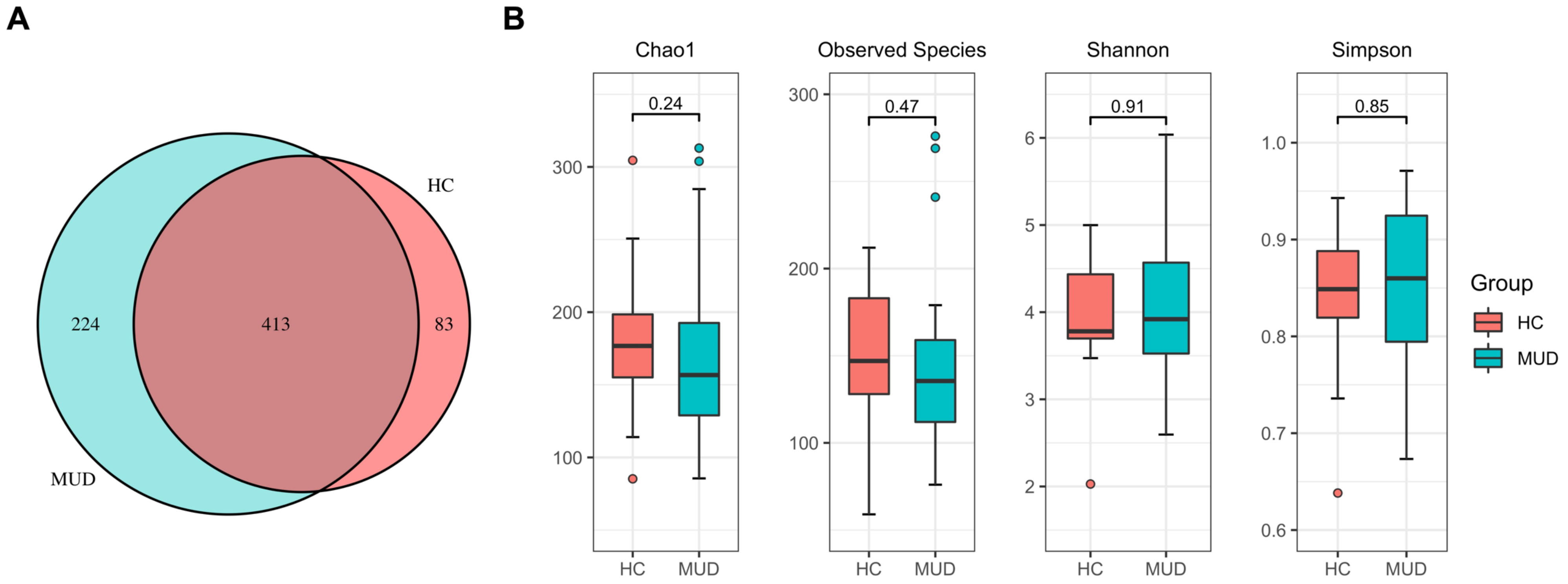

Supplementary Figure 3. Beta-diversity of the gut microbial communities in the MUD and HC groups. Principal component analysis (PCoA) plots based on **(A)** Bray-Curtis, **(B)** Jaccard, **(C)** unweighted UniFrac and **(D)** weighted UniFrac distance. PERMANOVA, all p > 0.05.

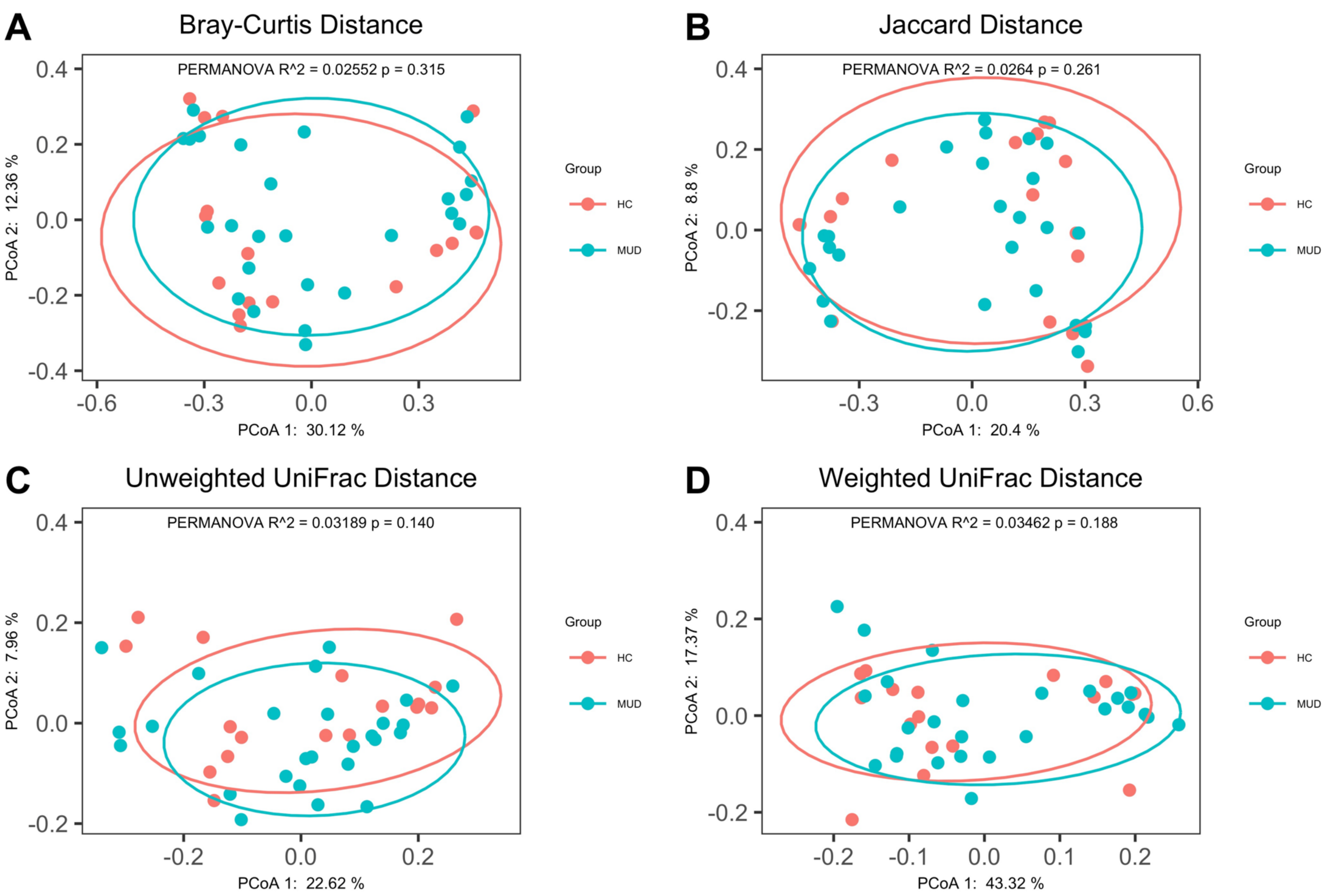

Supplementary Figure 4. Composition and relative abundance of taxa at multiple phylogenetic ranks in the HC and MUD groups based on 16S rRNA sequences. Relative abundance of major taxa at the level of phylum**(A)**, class**(B)**, order**(C)**, family**(D)** and genus**(E)** are presented in a stacked bar plot.

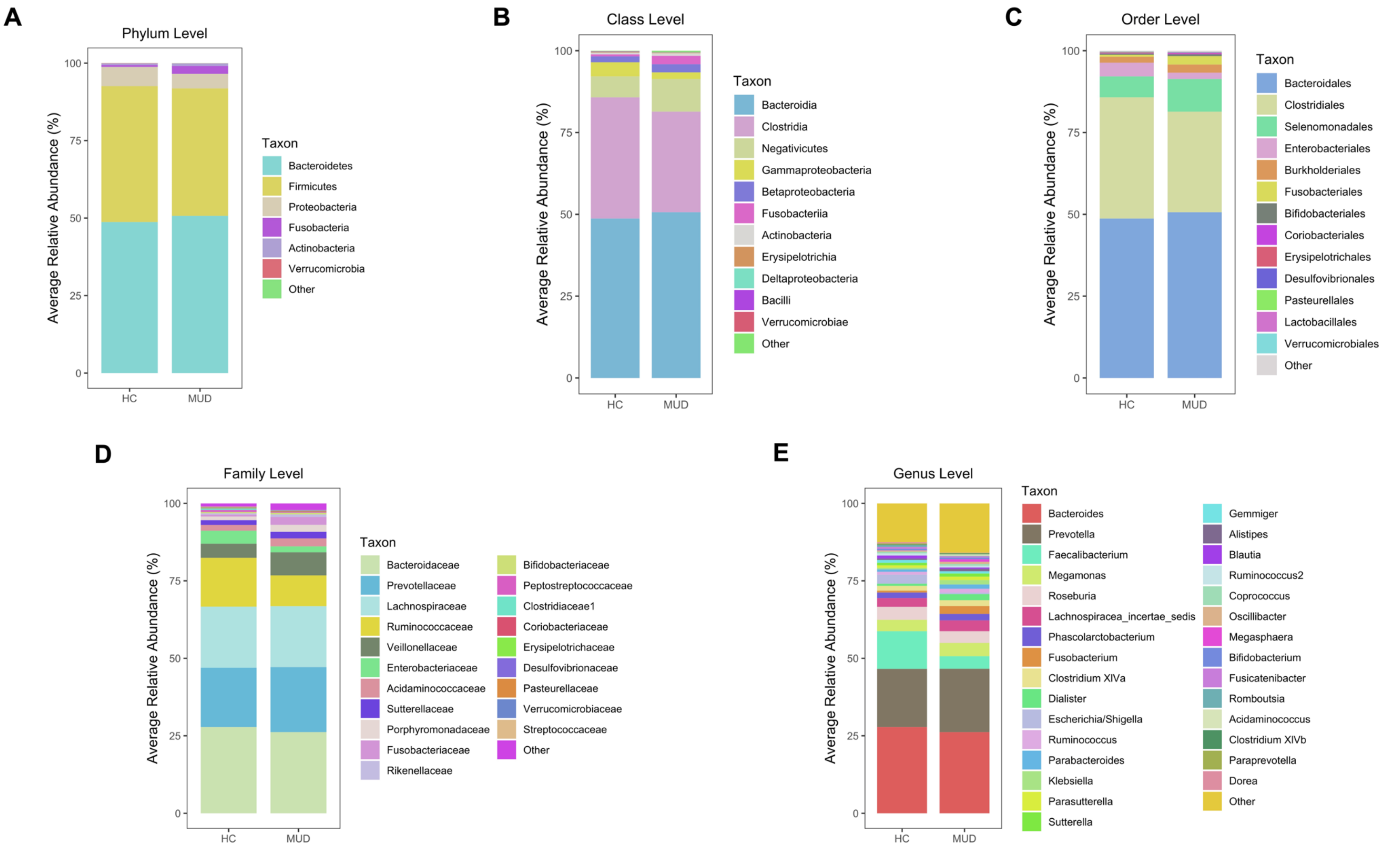

Supplementary Figure 5. Functional prediction for the fecal microbiome of the MUD and HC groups. The KOs with significantly different abundances identified using PICRUSTs are shown (Wilcoxon rank sum test, FDR q < 0.05). Bar plots represent the mean ± SEM of the abundance of the KOs.

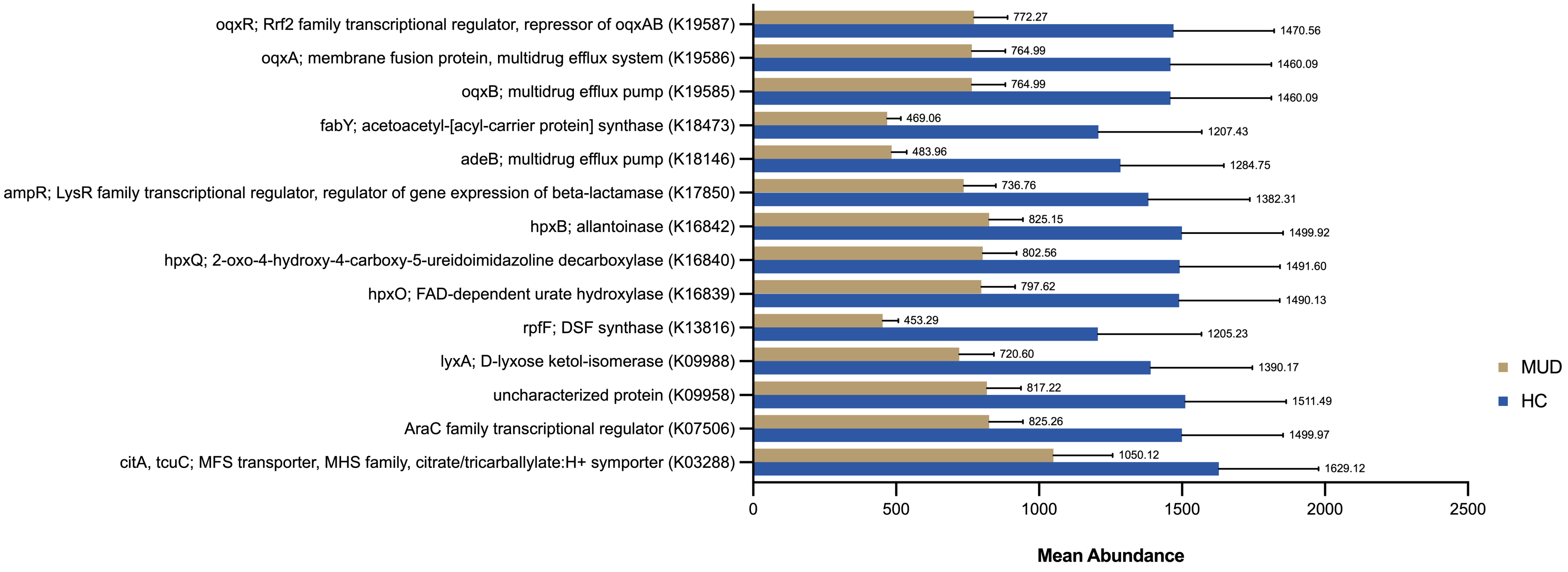

Supplementary Table 1. Association between covariates and microbial taxa.

Taxa	Age			BMI			AUDIT total score			FTND total score		
	Coef.[#]	p value	q value	Coef.[#]	p value	q value	Coef.[#]	p value	q value	Coef.[#]	p value	q value
p__Proteobacteria	0.0547	0.0073	0.1096	/	/	/	/	/	/	/	/	/
p__Actinobacteria	/	/	/	-0.0163	0.0340	0.2470	/	/	/	-0.0206	0.0230	0.2230
p__Firmicutes	/	/	/	/	/	/	/	/	/	-0.0606	0.0435	0.2470
p__Fusobacteria	/	/	/	/	/	/	/	/	/	0.0493	0.0494	0.2470
p__Proteobacteria; c__Gammaproteobacteria	0.0647	0.0051	0.1592	/	/	/	/	/	/	/	/	/
p__Proteobacteria; c__Gammaproteobacteria; o__Enterobacteriales	0.0683	0.0038	0.0822	/	/	/	/	/	/	/	/	/
p__Actinobacteria; c__Actinobacteria; o__Coriobacteriales	/	/	/	/	/	/	-0.0108	0.0056	0.0915	-0.0179	3.00E-05	0.0010
p__Proteobacteria; c__Gammaproteobacteria; o__Enterobacteriales; f__Enterobacteriaceae	0.0683	0.0038	0.1271	/	/	/	/	/	/	/	/	/
p__Actinobacteria; c__Actinobacteria; o__Coriobacteriales; f__Coriobacteriaceae	/	/	/	/	/	/	-0.0112	0.0052	0.1301	-0.0185	2.68E-05	0.0013
p__Proteobacteria; c__Gammaproteobacteria; o__Enterobacteriales; f__Enterobacteriaceae; g__Escherichia.Shigella	0.0551	0.0026	0.1425	/	/	/	/	/	/	/	/	/
p__Actinobacteria; c__Actinobacteria; o__Coriobacteriales; f__Coriobacteriaceae; g__Collinsella	/	/	/	/	/	/	-0.0130	0.0052	0.1908	-0.0188	0.0002	0.0201

p__Firmicutes; c__Clostridia; o__Clostridiales; f__Lachnospiraceae; g__Coprococcus	/	/	/	/	/	/	/	/	/	-0.0276	0.0034	0.1425
p__Firmicutes; c__Clostridia; o__Clostridiales; f__Lachnospiraceae; g__Lachnospiracea_incertae_sedis	/	/	/	/	/	/	/	/	/	-0.0598	0.0030	0.1425

[#] *The coefficients from the generalized linear model using MaAsLin2.*
